# GLP-1 enhances hyperpolarization-activated currents of mouse cerebellar Purkinje cell *in vitro*

**DOI:** 10.3389/fnmol.2023.1126447

**Published:** 2023-04-05

**Authors:** Yang Liu, Li-Xin Cao, Wei-Yao Wang, Yong-Rui Piao, Jun-Ya Wang, Chun-Ping Chu, Yan-Hua Bing, De-Lai Qiu

**Affiliations:** ^1^Department of Physiology and Pathophysiology, College of Medicine, Yanbian University, Yanji, Jilin, China; ^2^Department of Physiology, College of Basic Medicine, Jilin Medical University, Jilin, Jilin, China; ^3^Department of Urology, Affiliated Hospital of Yanbian University, Yanji, Jilin, China; ^4^Functional Experiment Center, College of Medicine, Yanbian University, Yanji, Jilin, China

**Keywords:** glucagon-like peptide-1 (GLP-1), cerebellar Purkinje cell, whole-cell patch-clamp recording, hyperpolarization-activated current (IH), protein kinase A

## Abstract

Glucagon-like peptide-1 (GLP-1) is mainly secreted by preglucagonergic neurons in the nucleus tractus solitarius, which plays critical roles in regulation of neuronal activity in the central nervous system through its receptor. In the cerebellar cortex, GLP-1 receptor is abundantly expressed in the molecular layer, Purkinje cell (PC) layer and granular layer, indicating that GLP-1 may modulate the cerebellar neuronal activity. In this study, we investigated the mechanism by which GLP1 modulates mouse cerebellar PC activity *in vitro*. After blockade of glutamatergic and GABAergic synaptic transmission in PCs, GLP1 increased the spike firing rate accompanied by depolarization of membrane potential and significantly depressed the after-hyperpolarizing potential and outward rectifying current of spike firing discharges *via* GLP1 receptors. In the presence of TTX and Ba^2+^, GLP1 significantly enhanced the hyperpolarized membrane potential-evoked instant current, steady current, tail current (I-tail) and hyperpolarization-activated (IH) current. Application of a selective IH channel antagonist, ZD7288, blocked IH and abolished the effect of GLP1 on PC membrane currents. The GLP1 induced enhancement of membrane currents was also abolished by a selective GLP1 receptor antagonist, exendin-9-39, as well as by protein kinase A (PKA) inhibitors, KT5720 and H89. In addition, immunofluorescence detected GLP1 receptor in the mouse cerebellar cortex, mostly in PCs. These results indicated that GLP1 receptor activation enhanced IH channel activity *via* PKA signaling, resulting in increased excitability of mouse cerebellar PCs *in vitro*. The present findings indicate that GLP1 plays a critical role in modulating cerebellar function by regulating the spike firing activity of mouse cerebellar PCs.

## 1. Introduction

In the mammalian central nervous system, GLP1 is mainly secreted by preglucagonergic neurons in the nucleus tractus solitarius (Larsen et al., 1997) that project widely to many regions of the brain, including the hypothalamus, amygdala, brain stem and cerebellum (Vrang et al., 2007; Llewellyn-Smith et al., 2011). GLP1 receptor is a G protein-coupled receptor that is widely expressed in the paraventricular nucleus, supraoptic nucleus, arcuate nucleus, hippocampus, amygdala, ventral tegmental area, substantia nigra, nucleus tractus solitarius, and cerebellum (Campos et al., 1994; Dunphy et al., 1998; Pyke et al., 2014; Cork et al., 2015; Heppner and Perez-Tilve, 2015; Jensen et al., 2018). GLP1 receptor is considered to be an excitatory G protein coupled receptor that plays critical roles in regulating neuronal activity and synaptic transmission (Hällbrink et al., 2001; Cork et al., 2015; Williams et al., 2018).

GLP1 receptor activation is involved in regulating multiple ion channels and signaling pathways, such as calcium channels, non-selective cation channels, voltage-dependent potassium channels and TRPC5 channels, and protein kinase A (PKA) and protein kinase C signaling pathways (Chen et al., 2021). GLP1 receptor activation can increase the level of cyclic adenosine monophosphate (cAMP) by activating adenylate cyclase, and subsequent activation of PKA. PKA signaling is a critical upstream component for many intracellular cytoplasmic signals, gene transcription, and protein synthesis responses that are implicated in neuronal activity and synaptic transmission (Mayo et al., 2003). Using whole-cell patch-clamp and microfluorescence techniques, GLP1 has been shown to induce depolarization of neuronal resting membrane potential and increase intracellular Ca^2+^ and cAMP concentration, resulting in increased spike firing frequency (Kakei et al., 2002). Application of GLP1 elicits inward currents, accompanied by increased membrane conductance in paraventricular nucleus corticotropin-releasing hormone neurons (Cork et al., 2015). GLP1 receptor activation increases intracellular Ca^2+^ concentration through a Gq/11 pathway and recruits β-Arrestin to promote ERK1/2 signal transduction (Wheeler et al., 1993; Koole et al., 2010). GLP1 receptor activation also inhibits adenosine 5′-monophosphate-activated protein kinase (AMPK) and activates mitogen-activated protein kinase (MAPK) in neurons through PKA signaling (Hayes et al., 2011) and inhibits AMPK in the hindbrain and activates MAPK *via* protein kinase C signaling, thereby participating in the regulation of animal food intake (Chen et al., 2021).

Under *in vitro* conditions, application of GLP-1 induces bi-directional membrane potential changes in neurons in the bed nucleus of striaterminalis through its receptor. GLP-1 regulates the opening of potassium channels which induces hyperpolarized membrane potential and an increase of membrane conductance (Williams et al., 2018). Activation of GLP-1 receptor can enhance the frequency of miniature excitatory postsynaptic currents (mEPSCs) through modulation of presynaptic AMPA/Kainate receptor, indicating that activation of GLP-1 receptor regulates the synthesis and release of glutamate (Mietlicki-Baase et al., 2014). Moreover, GLP1 receptor activation reduces the conductance of voltage-dependent potassium channels (Thiebaud et al., 2019), resulting in an increase in the spontaneous discharge frequency of olfactory granulosa cells (Cork et al., 2015). After blocking GABAergic synaptic transmission, stimulation of preglucagonergic neurons leads to increased excitation of mitral cells, indicating that preglucagonergic neurons are involved in shaping the discharge pattern of mitral cells (Thiebaud et al., 2019). GLP-1 induces excitation and leads to changes in the neuronal excitability of the olfactory cortex and hypothalamus region by inhibiting the conduction of voltage-dependent potassium channels and enhancing the release of glutamate (Schwartz et al., 2021).

PCs are the sole output of the cerebellar cortex and release GABA from their axons in the deep cerebellar nucleus (Ito, 1984, 2002). In addition to climbing fibers and parallel fibers, cerebellar PCs also receive adrenocorticotropic, monoaminergic and preglucagonergic projections, which are involved in the regulation of PC spike firing activity (Larsen et al., 1997; Merchenthaler et al., 1999). PCs exhibit intrinsic simple spike activity, which is dependent on activation of the persistent sodium current, IH, and voltage-dependent potassium current (Ito, 1984, 2006; Libster et al., 2015; Light et al., 2015). Activation of G protein-coupled receptors can increase the IH in mouse cerebellar PCs, resulting in an increased simple spike firing rate *in vitro* (Libster et al., 2015). GLP1 receptor is abundant in cerebellar PCs (Hamilton and Hölscher, 2009) and the cerebellar cortex receives preglucagonergic neuronal projections (Campos et al., 1994; Cork et al., 2015), indicating that GLP1 receptor activation may regulate motor function and coordination by regulating the output of cerebellar PCs. However, the effect of GLP1 on cerebellar PC activity is unclear. We therefore investigated the mechanism by which GLP1 affects PC simple spike firing activity using whole-cell patch-clamp recording, immunohistochemistry and neuropharmacology methods.

## 2. Materials and methods

### 2.1. Slice preparation

The experimental procedures were approved by the Animal Care and Use Committee of Yanbian University and were in accordance with the animal welfare guidelines of the U.S. National Institutes of Health. The permit number is SYXK (Ji) 2011–006. Cerebellar slices preparation has been previously described (Xuan et al., 2018). In short, after deeply anaesthetization with halothane, adult (4˗6 weeks old) ICR (Institute of Cancer Research) mice were decapitated immediately. The cerebellum was sectioned and placed in ice-cold artificial cerebrospinal fluid (ACSF) containing (in mM): 125NaCl, 3KCl, 1 MgSO_4_, 2 CaCl_2_, 1.25 Na_2_HPO_4_, 25 NaHCO_3_, and 10 D-glucose bubbled with 95% O_2_ and 5% CO_2_ (pH 7.4; 295–305 mOsm). The sagittal slices of cerebellar cortex (300 μm thick) were prepared using a Vibratome (VT 1200s, Leica, Nussloch, Germany). The slices were incubated at least 1 h in a submerged chamber filled with 95%O_2_ and 5% CO_2_ equilibrated ACSF at room temperature (24–25°C) prior to recording.

### 2.2. Electrophysiological recordings

Whole-cell patch-clamp recordings from cerebellar PCs were visualized using a 60x water-immersion lens through a Nikon microscopy (Eclipse FN1, Nikon Corp., Tokyo, Japan). Patch electrodes contained a solution of the following (in mM): potassium gluconate 120, HEPES 10, EGTA 1, KCl 5, MgCl2 3.5, NaCl 4, biocytin 8, Na_2_ATP 4, and Na_2_GTP 0.2 (pH 7.3 with KOH, osmolarity adjusted to 300 mOsm). Patch pipette resistances were 4˗6 MΩ in the bath, with series resistances in the range of 10–20 MΩ. Membrane potentials and/or currents were monitored with an Axopatch 700B amplifier (Molecular Devices, Foster City, CA, United States), filtered at 5 kHz, and acquired through a Digidata 1,440 series analog-to-digital interface on a personal computer using Clampex 10.4 software (Molecular devices, Foster City, CA, United States). Under voltage clamp recording mode, cells were held in voltage-clamp mode at −70 mV. Series resistance was monitored by applying voltage pulses (10 ms, 5 mV), and only neurons with stable series resistance were include in the analysis. SR95531 (10 μM) and kynurenic acid (KYC; 1 mM) were included in the ACSF to block the GABAergic and glutamatergic synaptic transmission. TTX (500 nM) and BaCl_2_ (100 μM) were added to the ACSF to block the voltage gated sodium channel and Ba^2+^-sensitive potassium channel, so as to separate the IH current (Cardenas et al., 1999; Qiu et al., 2003). IH current is activated by hyperpolarization current or membrane potential. For some experiments under current-clamp mode, the membrane potential was hyperpolarized to −80 mV by injecting negative currents (−20 to −30 pA) under control conditions.

### 2.3. Immunohistochemistry and imaging

Mice (*n* = 4) were deeply anesthetized with an intraperitoneal injection of 7% chloral hydrate (5 ml/kg), and then transcardially perfused by cold phosphate buffer (PBS) at pH 7.4, followed by 4% paraformaldehyde (PFA, Sinopharm Chemical Reagent Co., China) PBS solution. Brain was post-fixed in 4% PFA for 48 h at 4°C, and washed with PBS 5 times at 10 min intervals. Separate the cerebellum from the brain with a razor blade. The cerebellum was exposed to 10% sucrose, 20% sucrose and 30% sucrose in PBS for more than 6 h. After embedding in Tissue-Tek O.C.T. Compound (Beijing zhongshan Jin qiao Biotechnology Co, China), the cerebellum was quickly frozen in −80°C refrigerator for 2 h. Then cerebellum was sectioned into 8 μm slices in the sagittal plane using a freezing microtome (CM1900, Leica, Germany). Slices were stored at 4°C for immunohistochemical experiments.

Microscope slides were permeabilized with 0.3% Triton X-100 in PBS, and then were blocked (10% donkey serum in PBS) and incubated in a primary antibody (rabbit anti-GLP-1 receptor, 1:1,000, Sigma-Aldrich), followed by Cy3 labeled goat anti-rabbit antibody (Life Tech, 1:200) and 4′,6-diamidino-2-phenylindole (DAPI, 1:1,000). The incubation time for the primary antibody was overnight at 4°C. For the secondary antibody and DAPI, the incubation time was 2 h at room temperature. Microscope slides with slices were then washed 3 times in PBS covered with a coverslip and sealed with nail polish. Fluorescence images were acquired by confocal laser-scanning microscope (Nikon C2, Tokyo, Japan) (Wang et al., 2018; Wu et al., 2020). A large region images including the desired region was obtained on Nikon C2 laser confocal system with 10X objective.

### 2.4. Drug application

GLP-1 (100 nM), Exendin 9–39 (100 nM), Exendin-4 (100 nM), TTX (500 nM), SR95531 (10 μM), kynurenic acid (KYC; 1 mM), ZD7288 (50 μM), KT5720 (500 nM), and H89 (10 μM) were used in the experiments. These drugs were bought from Sigma-Aldrich (Shanghai, China). All drugs were dissolved in solution and kept in frozen in aliquots, and were applied to the cerebellar slices at 0.5 ml/min in ACSF through the peristaltic pump. In the experiments involving PKA inhibitor, application of KT5720 or H89 was began at least 20 min before patch-clamp recording and continuing throughout the experiments.

### 2.5. Statistical analysis

Electrophysiological data were analyzed using Clampfit 10.3 software. All data are expressed as the mean ± S.E.M. IH was determined by subtracting I-Instant from I-steady at each hyperpolarizing voltage step using the equation: IH = I-steady – I-instant. Student’s paired t-test (SPSS software) was used to determine the level of statistical significance between groups of data. *p-*values below 0.05 were considered to indicate a statistically significant difference between experimental groups.

## 3. Results

### 3.1. GLP1 enhances the excitability of mouse cerebellar PCs *via* its receptor *in vitro*

**Figure 1:**
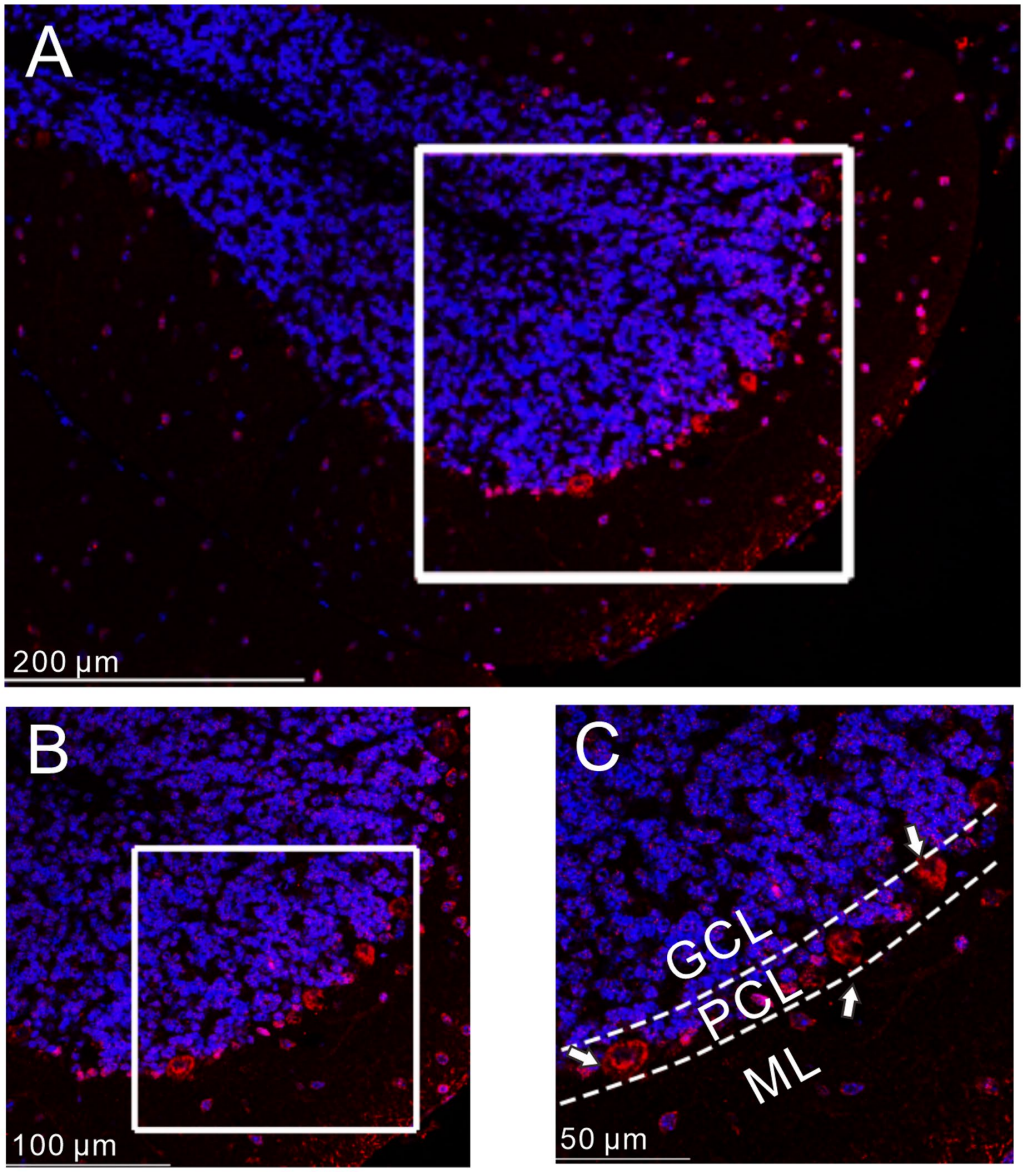
Immunofluorescence of GLP-1 receptor was expressed in cerebellar PCs. **(A)** A digital micrograph shows the confocal image of DAPI (blue) and GLP-1 receptor (red) in cerebellar cortex. DAPI (blue) was used for neuronal soma location. **(B)** Magnified image of the box area in **(A)**. **(C)** Enlarged image of the box area in **(B)**. The white arrows indicate the GLP-1 receptor expressed in the PCs. ML, molecular layer; PCL, Purkinje cell layer; GCL, granular cell layer.

We first examined the distribution of GLP1 receptor in the mouse cerebellar cortex using immunohistochemistry. As shown in Figure 1, GLP1 receptor was present in the cerebellum and was most concentrated in the PC layer. This indicates that GLP1 receptor is expressed in mouse cerebellar PCs.

**Figure 2:**
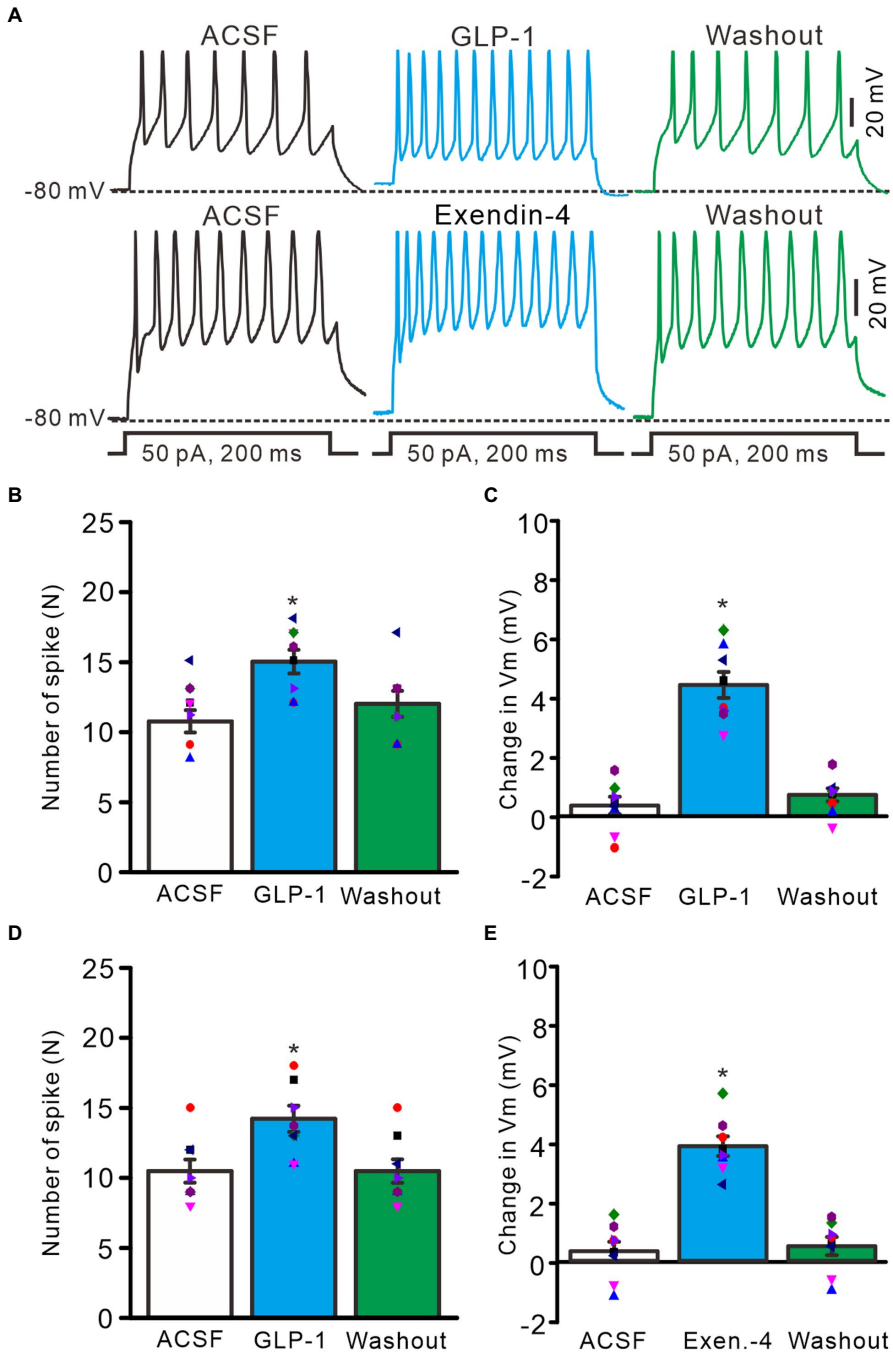
GLP-1 and Exendin-4 enhanced excitability of cerebellar PCs. **(A)** Under current-clamp recording mode (Vm = −80 mV), the representative traces showing the spike firing elicited by depolarization currents (50 pA, 200 ms) during treatments with ACSF, GLP-1 (100 nM; upper), Exendin-4 (100 nM; lower) and washout. **(B)** The mean (± S.E.M) with individual data showing the number of spikes evoked by depolarization current during treatments with ACSF, GLP-1 and washout. **(C)** The mean (± S.E.M) with individual data showing the change of resting membrane potential (Vm) during treatments with ACSF, GLP-1 and washout. **(D)** The mean (± S.E.M) with individual data showing the number of spikes evoked by depolarization current during treatments with ACSF, Exendin-4 and washout. **(E)** The mean (±S.E.M) with individual data showing the change of resting membrane potential during treatments with ACSF, Exendin-4 and washout. *p < 0.05 vs. ACSF, *n* = 8 cells in each group.

Perfusion of GLP1 (100 nM) increased the membrane excitability of PCs, which was manifested by a significant increase in the frequency of spike firing discharge elicited by depolarization current, accompanied by significant depolarization of membrane potential (Figure 2A). After administration of GLP1 (100 s), the number of spike firing discharge induced by depolarization current (50 pA, 200 ms) was 15.0 ± 0.85, which was significantly higher than that in the ACSF group (ACSF: 10.75 ± 0.80; *p* < 0.05, *n* = 8, Figure 2B). The GLP1-induced depolarization of membrane potential was 4.45 ± 0.44 mV, which was significantly different than that in the ACSF group (ACSF: 0.36 ± 0.29 mV; *p* < 0.05, *n* = 8, Figure 2C). To clarify whether GLP1 receptor activation can increase the excitability of PCs, we used the GLP1 receptor agonist, exendin-4 (100 nM). Perfusion of exendin-4 (100 nM) increased the frequency of spike firing (Figure 2C). In the presence of exendin-4 (100 s), the frequency of spike firing evoked by depolarization current (50 pA, 200 ms) was 14.25 ± 0.91, which was significantly higher than that in the ACSF group (ACSF: 10.5 ± 0.82; *p* < 0.05, *n* = 8, Figure 2D). GLP1 induced depolarization of membrane potential was 3.93 ± 0.33 mV, which was significantly different than that in the ACSF group (*p* < 0.05 versus ACSF, *n* = 8, Figure 2E). These results indicate that GLP1 receptor activation enhances the excitability of PCs.

**Figure 3:**
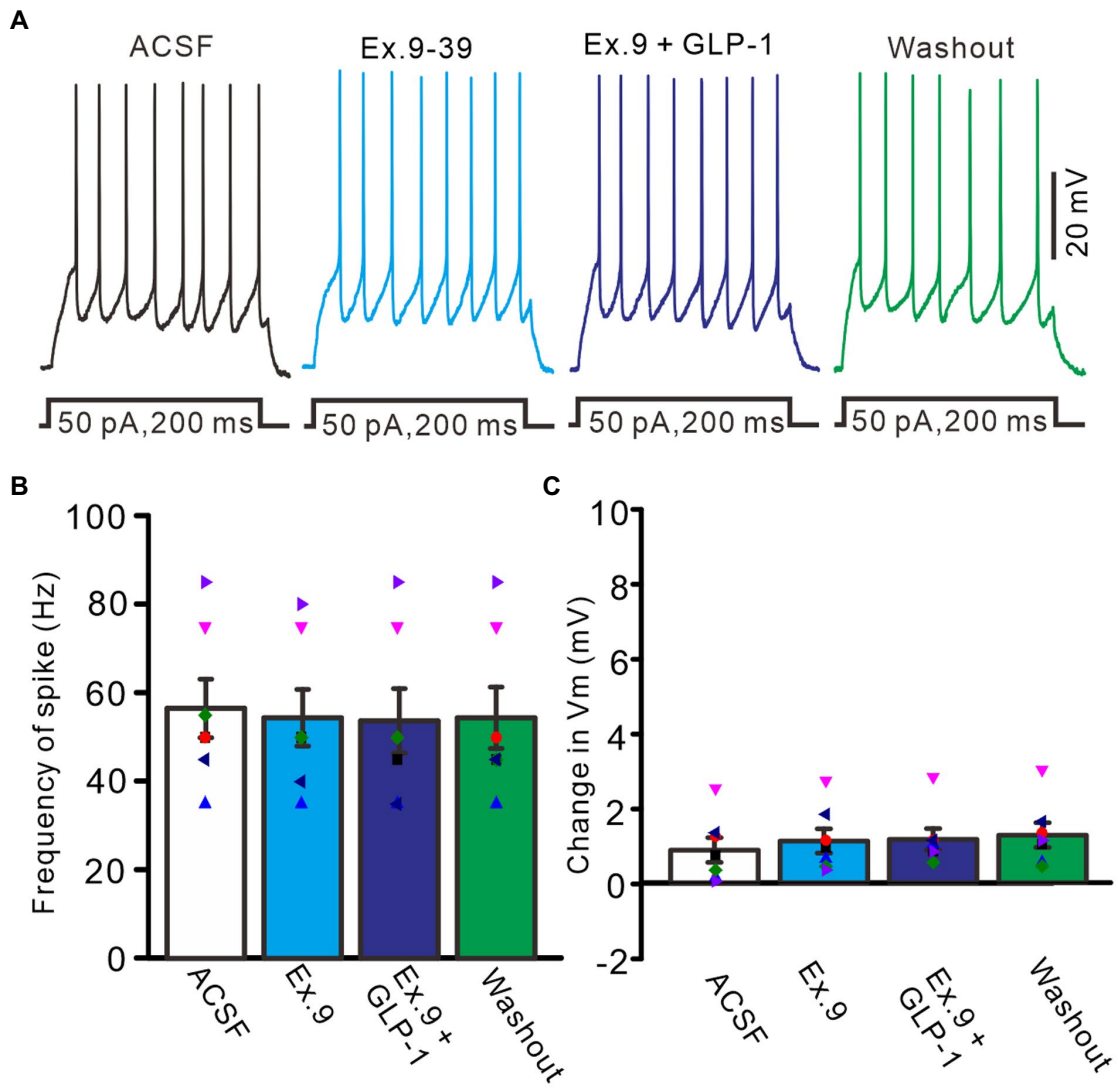
Blockade of GLP-1 receptor, GLP-1 failed to enhance the excitability of PCs. **(A)** Under current-clamp recording mode (Vm = −80 mV), the representative traces showing PCs in response to depolarization currents (50 pA, 200 ms) during treatments with ACSF, GLP-1 (100 nM), Exendin-39 (Ex. 39; 100 nM), Exendin-39 + GLP-1 (100 nM) and washout. **(B)** The mean (± S.E.M) with individual data showing the number of spikes evoked by depolarization current during treatments with ACSF, Exendin-39 (Ex. 39), Exendin-39 + GLP-1 and washout. **(C)** The mean (± S.E.M) with individual data showing the change of resting membrane potential during treatments with ACSF, Exendin-39 (Ex. 39), Exendin-39 + GLP-1 and washout.

We then used a GLP1 receptor blocker, exendin-9-39 (100 nM), to determine whether GLP1 increased the number of spike discharges by PCs through its receptor. Perfusion of exendin-9-39 had no significant effect on PC excitability, but completely blocked the GLP1-induced increase in spike discharges (Figure 3A). In the presence of exendin-9-39 (100 nM), the number of spike discharges elicited by depolarization current (50 pA, 200 ms) was 10.9 ± 1.28, which was similar to that in the ACSF group (*p* > 0.05, *n* = 7, Figure 3B), and the change in membrane potential was 1.11 ± 0.32 mV, which was not significantly different from that in the ACSF group (0.87 ± 0.33 mV; *p* > 0.05, *n* = 7, Figure 3C). In the presence of exendin-9-39 and GLP1, the number of spike discharges induced by depolarization current (50 pA, 200 ms) was 10.7 ± 1.46, which was similar to that in the ACSF group (*p* > 0.05, *n* = 7, Figure 3B). The change in membrane potential was 1.15 ± 0.29 mV, which was not significantly different from that in the ACSF group (0.87 ± 0.33 mV; *p* > 0.05, *n* = 7, Figure 3C). These results indicate that GLP1 activates its receptors on PCs, resulting in membrane depolarization and increased frequency of spike discharge.

**Figure 4:**
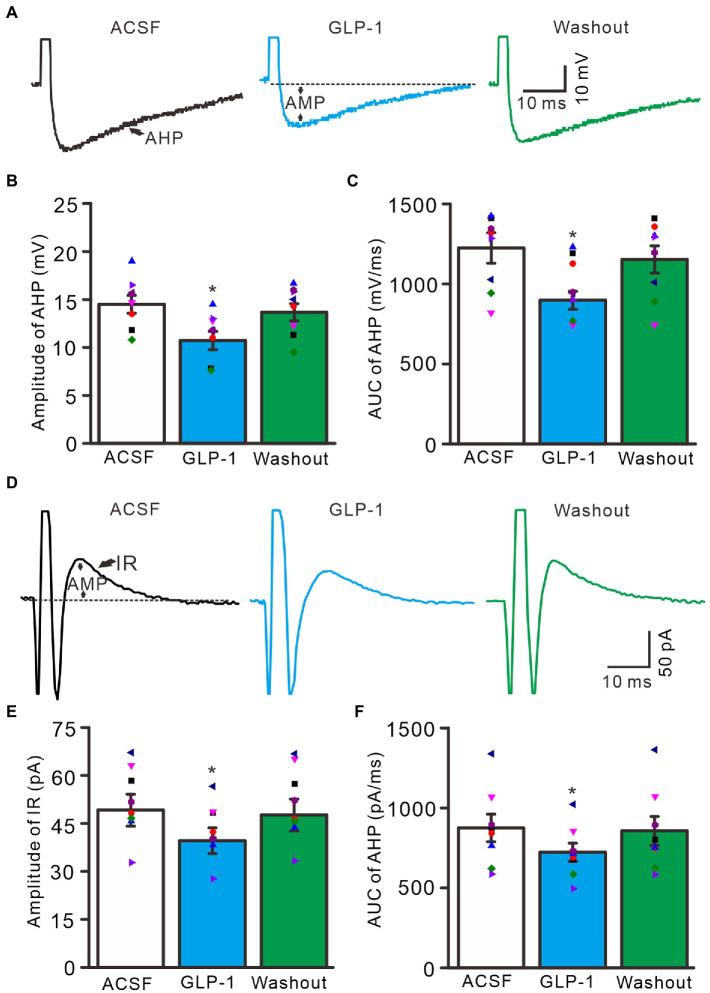
Effect of GLP-1 on after-hyperpolarization (AHP) of the depolarization currents-evoked action potential. **(A)** Representative traces showing AHP of the depolarization currents (10 pA, 1 ms)-evoked action potentials during treatments of ACSF, GLP-1 (100 nM), and washout. **(B)** The mean (± S.E.M) with individual data showing the amplitude of AHP during treatments with ACSF, GLP-1 and washout. **(C)** The mean (± S.E.M) with individual data showing the area under the curve (AUC) of AHP during each treatment. **(D)** Representative traces showing outward rectified current (IR) of the evoked simple spike discharge during each treatment. **(E)** The mean (± S.E.M) with individual data showing the amplitude of IR during each treatment. **(F)** The mean (± S.E.M) with individual data showing AUC of IR during each treatment. *p < 0.05 vs. ACSF, *n* = 8 cells in each group.

Under current-clamp recording conditions, GLP1 significantly depressed after-hyperpolarization potential, resulting in significantly reduced amplitude and area under the curve (AUC) (Figure 4A). In the presence of GLP1, after-hyperpolarization potential amplitude was 10.7 ± 0.96 mV, which was significantly lower than that of baseline (ACSF: 14.5 ± 0.93 mV; *p* < 0.05, *n* = 8, Figure 4B), and the AUC of the after-hyperpolarization potential was 897.6 ± 56.0 mV/ms, which was significantly different from that of baseline (ACSF: 1225.3 ± 95.0 mV/ms; *p* < 0.05, *n* = 8, Figure 4C). In voltage-clamp recording mode, GLP1 significantly depressed the amplitude of the outward rectified current (IR), resulting in decreased amplitude and AUC of IR (Figure 4D). In the presence of GLP1, the IR amplitude was 39.6 ± 4.0 pA, which was significantly lower than that of baseline (ACSF: 49.1 ± 5.0 pA; *p* < 0.05, *n* = 8, Figure 4E); and the AUC of the IR was 722.2 ± 57.0 pA/ms, which was significantly different than that of baseline (ACSF: 874.2 ± 86 pA/ms; *p* < 0.05, *n* = 8, Figure 4F). These results indicate that GLP1 depressed the after-hyperpolarization potential of the action potentials in cerebellar PCs.

### 3.2. GLP-1 facilitated a hyperpolarization-activated cationic current (IH)

IH plays a key role in controlling the periodicity of continuous and intermittent action potential firing (Maccaferri and McBain, 1996; Ghamari-Langroudi and Bourque, 2000). IH channels are expressed in cerebellar PCs and participate in modulating simple spike firing activity (Crepel and Penit-Soria, 1986; Roth and Häusser, 2001) and in controlling the steady state of membrane potential (Southan et al., 2000). Activation of G protein-coupled receptors can regulate the activities of IH channels through cAMP, cGMP and/or Ca^2+^ signaling pathways, thereby modulating the excitability of neurons (Pape, 1996; Ludwig et al., 1998; Biel et al., 2002; Qiu et al., 2003). To understand the effect of GLP1 on IH activity, we examined the effect of GLP1 on IH channel activity.

**Figure 5:**
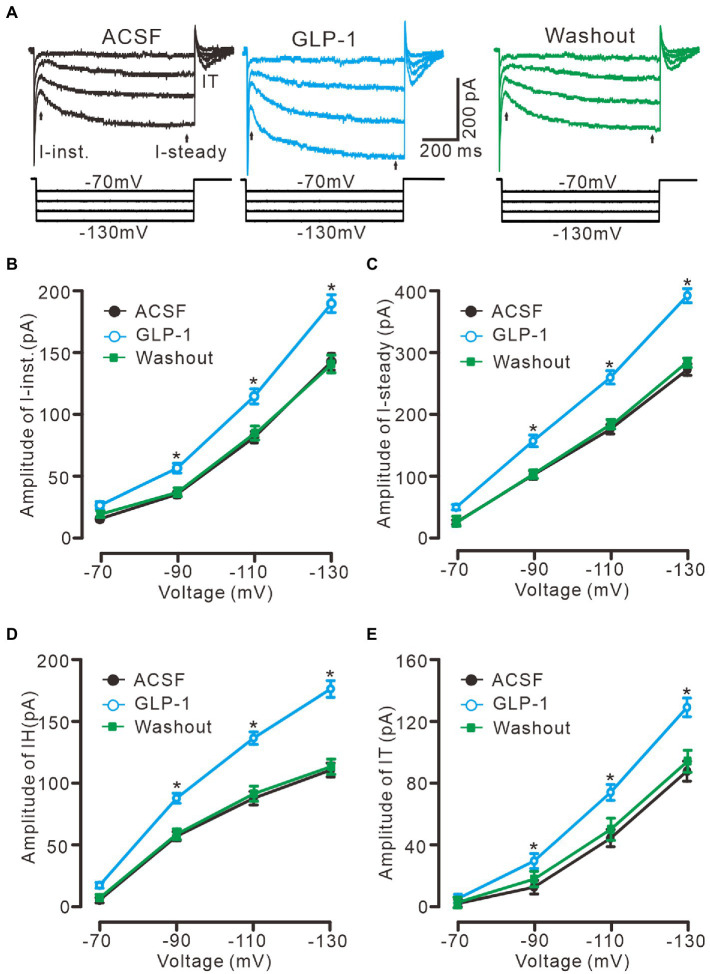
GLP-1 enhanced IH currents in cerebellar PCs. **(A)** Under voltage-clamp recording mode (V_hold_ = −60 mV), the representative traces showing membrane current traces elicited by a set of hyperpolarizing voltage step (20 mV decrements, 1 s) during treatments of ACSF, GLP-1 (100 nM) and washout. **(B,C)** The mean (± S.E.M) amplitude of the instant current (I-inst. **B**) and the steady state current (I-steady, C) evoked by hyperpolarizing steps during treatments with ACSF, GLP-1 and washout. **(D,E)** The mean (± S.E.M) amplitude of the IH **(D)** and the tail current (IT; **E**) evoked by hyperpolarizing steps during each treatment. *p < 0.05 vs. ACSF, *n* = 6 cells in each group.

In the presence of TTX (500 nM) and BaCl_2_ (100 μM), application of a series of hyperpolarized membrane potentials (−70 to −130 mV, 20 mV/step, 1 s) induced three kinds of membrane current, instant current (I-instant), steady current, and I-tail, which were significantly enhanced by perfusion of GLP1 (100 nM, Figure 5A). The GLP1 induced enhancement of I-instant (Figure 5B), I-steady (Figure 5C) and IH (I-steady − I-instant; Figure 5D) was evoked by hyperpolarizing membrane potential. GLP1 also significantly enhanced I-tail, which reflected the enhancement of IH (Figure 5E). These results indicated that GLP1 facilitates the IH in cerebellar PCs.

**Figure 6:**
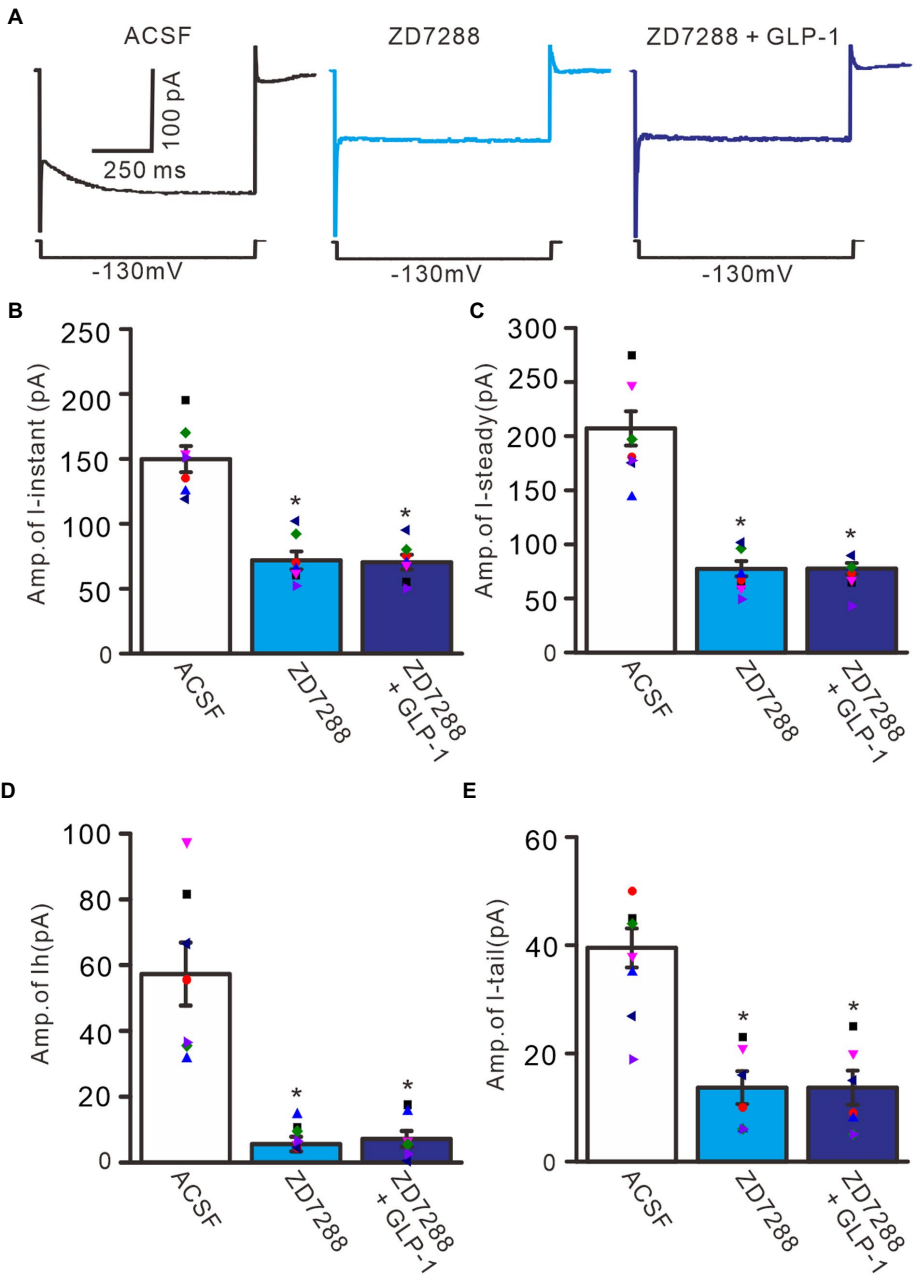
Blockade of IH abolished the effect of GLP-1 on membrane currents in PCs. **(A)** Under voltage-clamp recording mode (Vh = −60 mV), the representative traces showing membrane currents elicited by hyperpolarizing voltage steps (−130 mV, 1 s) during treatments of ACSF (control), ZD7288 (50 μM), and ZD7288 + GLP-1. **(B,C)** Bar graphs show that the mean (± S.E.M) and individual amplitude of I-instant (I-inst.; **B**) and I-steady **(C)** during treatments with ACSF, ZD7288 (50 μM), and ZD7288 + GLP-1. **(D,E)** Bar graphs show that the mean (± S.E.M) and individual amplitude of IH **(D)** and IT **(E)** during each treatment. *p < 0.05 vs. ACSF, *n* = 7 cells in each group.

To confirm that GLP1 enhanced the activity of the IH channel, we employed an IH channel blocker, ZD7288 (50 μM) and then observed the effect of GLP1 on the hyperpolarizing potential-evoked membrane current (Figure 6). Administration of GLP1 significantly enhanced the I-instant, I-steady, IH current and I-tail evoked by hyperpolarizing potential (−130 mV, 1 s). Application of ZD7288 blocked IH, and abolished the GLP1-enhanced membrane current (Figure 6A). In the presence of ZD7288, the I-instant was 71.9 ± 6.9 pA, which was significantly different than that of the ACSF group (149. 9 ± 10.1 pA; *p* < 0.05, *n* = 7, Figure 6B). Additional application of GLP1 failed to increase the I-instant. In the presence of ZD7288 and GLP1, the I-instant was 70.4 ± 5.7 mV, which was not significantly different from that of ZD7288 alone (*p* > 0.05, *n* = 7, Figure 6B). In the presence of ZD7288, the I-steady induced by hyperpolarization stimulation was 77.4 ± 7.0 pA, which was significantly different from that of the ACSF group (207.1 ± 15.8 pA; *p* < 0.05, *n* = 7, Figure 6C). Additional application of GLP1 failed to increase the I-steady. In the presence of ZD7288 and GLP1, the I-steady was 77.6 ± 5.2 pA, which was not significantly different from that of ZD7288 alone (*p* > 0.05, *n* = 7, Figure 6C). In the presence of ZD7288 and GLP1, IH was 7.1 ± 2.4 pA, which was not significantly different from that with ZD7288 alone (5.6 ± 2.2 pA; *p* > 0.05, *n* = 7, Figure 6D). In the presence of ZD7288 and GLP1, I-tail was 13.7 ± 3.0 mV, which was similar to that with ZD7288 alone (13.6 ± 8.3 pA; *p* > 0.05, *n* = 7, Figure 6E). These results indicate that blockade of IH channels can abolish the GLP1-induced enhancement of the membrane current in cerebellar PCs, indicating that GLP1 enhances the activity of IH channels and increases the excitability of PCs through GLP1 receptors.

**Figure 7:**
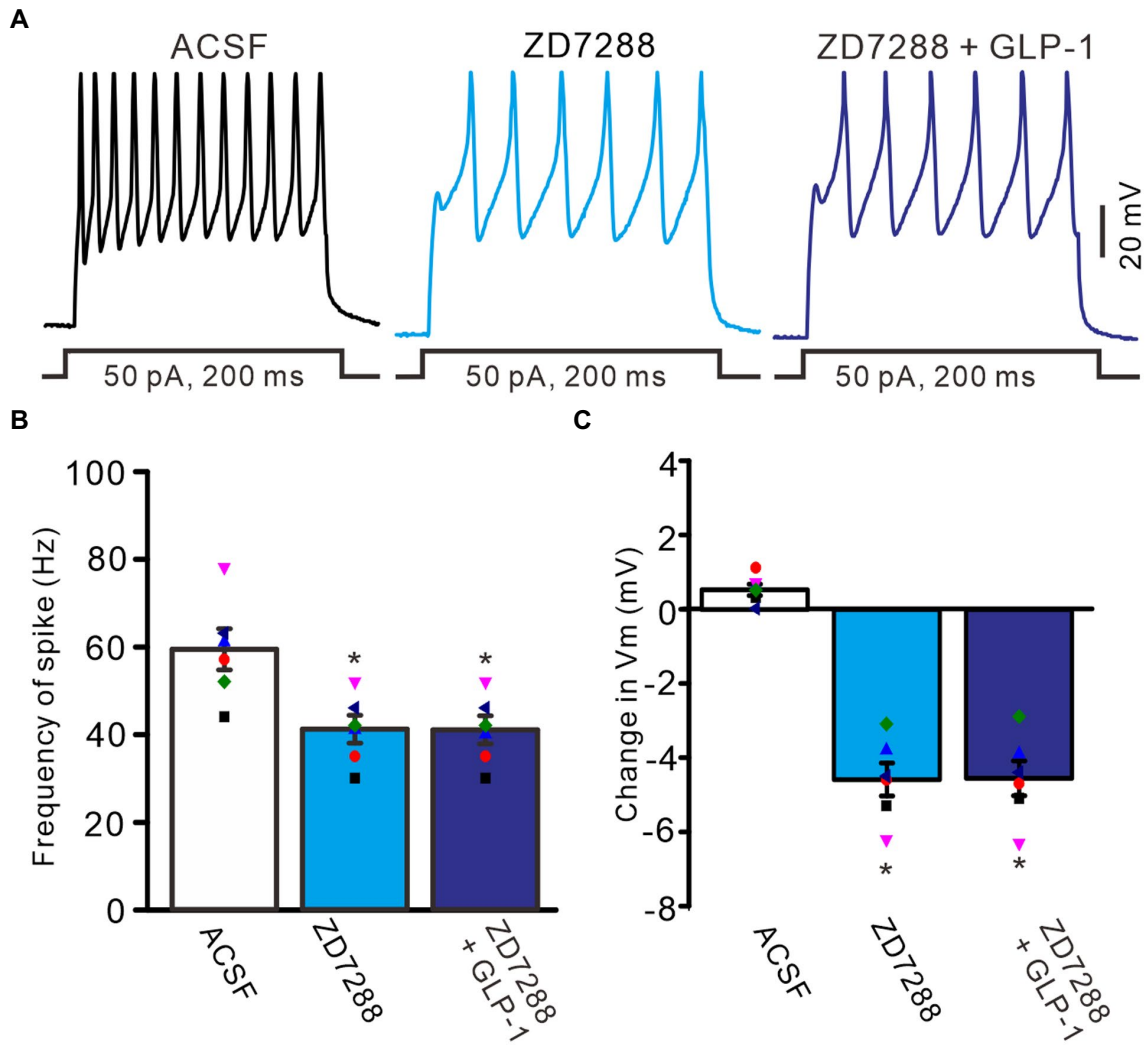
Blockade of IH, GLP-1 failed to enhance the excitability of PCs. **(A)** Under current-clamp recording mode (Vm = −80 mV), the representative traces showing PCs in response to depolarization currents (50 pA, 200 ms) during treatments of ACSF, ZD7288 (50 μM), and ZD7288 (50 μM) + GLP-1 (100 nM). **(B)** The mean (± S.E.M) with individual data showing the number of spikes evoked by depolarization current during each treatment. **(C)** The mean (± S.E.M) with individual data showing the change of resting membrane potential during each treatment. *p < 0.05 vs. ACSF, *n* = 6 cells in each group.

To understand the GLP1-induced depolarization through activation of hyperpolarization-activated cationic channels, we observed the effect of GLP1 on the excitability of PCs in the presence of ZD7288 (50 μM). Application of ZD7288 significantly decreased the excitability of PCs, which showed a significant decrease in the number of spike discharges elicited by depolarization current, accompanied by significant hyperpolarization of membrane potential (Figure 7A). In the presence of ZD7288, the number of spike discharges elicited by depolarization current (50 pA, 200 ms) was 8.0 ± 0.77, which was significantly lower than that in the ACSF group (11.8 ± 1.11; *p* < 0.05, *n* = 6, Figure 7B), and the change in membrane potential was −4.58 ± 0.45 mV, which was significantly lower than that in the ACSF group (0.54 ± 0.17 mV; *p* < 0.05, *n* = 6, Figure 7C). Notably, IH blockade abolished the effect of GLP1 on PC excitability. In the presence of ZD7288 and GLP1 (100 nM), the number of spike firing discharges elicited by depolarization current was 8.2 ± 0.77, which was not significantly different than that with 7,288 alone (ACSF; *p* < 0.05, *n* = 6, Figure 7B). The change in membrane potential was −4.55 ± 0.47 mV, which was significantly lower than that in the ACSF group (0.54 ± 0.17 mV; *p* < 0.05, *n* = 6), but it was not significantly different from that with ZD7288 alone (*p* > 0.05, *n* = 6, Figure 7C). These results indicated that IH blockade abolished the GLP1-induced depolarization of membrane potential, and that IH underlies GLP1-induced depolarization in mouse cerebellar PCs.

### 3.3. GLP1 facilitated IH through its receptor and PKA signaling

**Figure 8:**
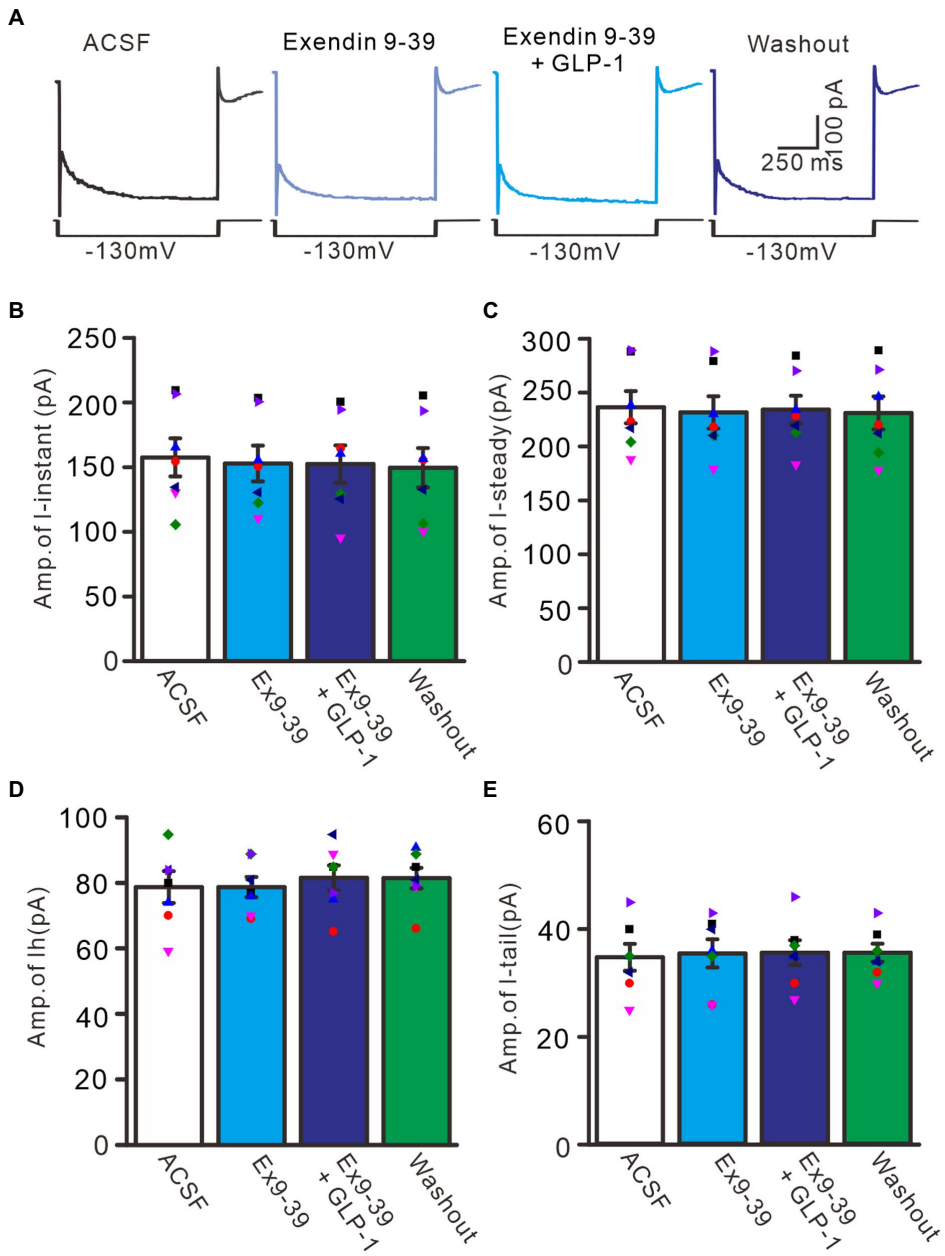
Blockade of GLP-1 receptor abolished the effect of GLP-1 on membrane currents in PCs. **(A)** Under voltage-clamp recording mode (Vh = −60 mV), the representative traces showing membrane currents elicited by hyperpolarizing voltage steps (−130 mV, 1 s) during treatments of ACSF, Exendin 9–39 (100 nM), Exendin9-39+ GLP-1, and washout. **(B,C)** Bar graphs show the mean (± S.E.M) and individual amplitude of I-instant (I-inst.; **B**) and I-steady **(C)** during each treatment. **(D,E)** Bar graphs show that the mean (± S.E.M) and individual amplitude of IH **(D)** and IT **(E)** during each treatment. *n* = 7 cells in each group.

We further employed the GLP1 receptor blocker, exendin-9-39 (100 nM), to examine whether GLP1 enhanced IH channel activity *via* its receptor. Administration of exendin-9-39 did not significantly affect I*-*instant, I-steady, IH, or I-tail induced by hyperpolarization potential (−130 mV, 1 s). In the presence of exendin-9-39, the mean I-instant was 152.9 ± 13.9 pA (*p* > 0.05 versus ACSF group, *n* = 7, Figures 8A,B), the mean I-steady was 231.6 ± 14.9 pA (*p* > 0.05 versus ACSF group, *n* = 7, Figure 8C), the mean IH was 78.7 ± 3.1 (*p* > 0.05 versus ACSF group, *n* = 7, Figure 8D), and the mean I-tail was 35.3 ± 2.6 pA (*p* > 0.05 versus ACSF, *n* = 7, Figure 8E). Moreover, application of exendin-9-39 completely blocked the effect of GLP1 on the hyperpolarization-elicited membrane current. In the presence of exendin-9-39 and GLP1, the mean I-instant was 152.4 ± 14.4 pA (*p* > 0.05 versus exendin-9-39 alone, *n* = 7, Figure 8B), the mean I-steady was 231.6 ± 14.9 pA (*p* > 0.05 versus exendin-9-39 alone, *n* = 7, Figure 8C), the mean IH was 81.6 ± 3.8 pA (ACSF) (*p* > 0.05 versus exendin-9-39 alone, *n* = 7, Figure 8D), and the mean I-tail was 35.4 ± 2.6 pA (*p* > 0.05 versus exendin-9-39 alone, *n* = 7, Figure 8E). These results indicate that GLP1 enhances the activity of IH channels and increases the excitability of PCs by activating GLP1 receptors.

**Figure 9:**
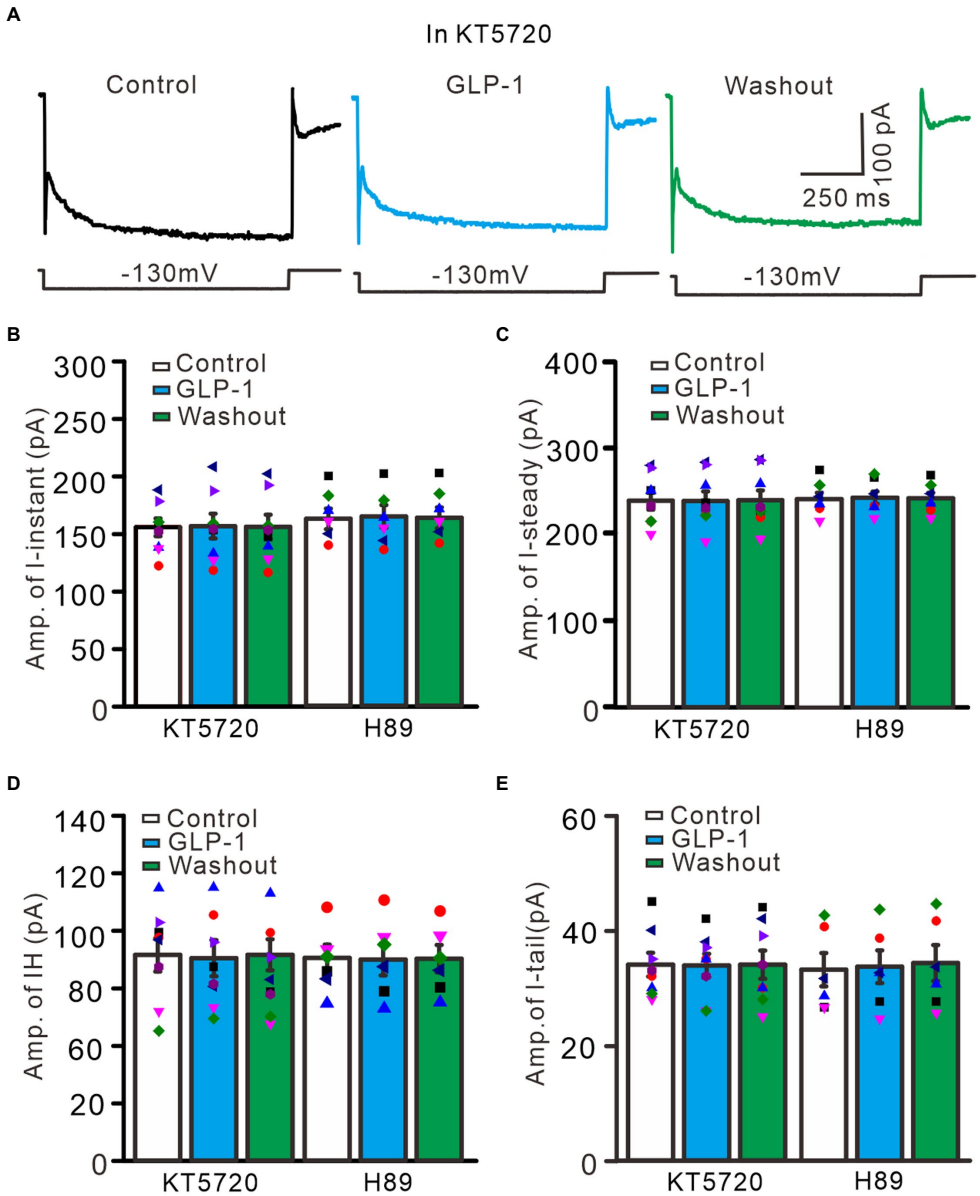
Inhibition of PKA, GLP-1 failed to enhance the hyperpolarizing voltage step-elicited membrane currents in PCs. **(A)** The representative traces showing membrane currents elicited by a hyperpolarizing voltage step (−130 mV, 1 s) during treatments with KT5720 (500 nM), KT5720 + GLP-1 (100 nM), and washout of GLP-1. **(B,C)** Left panel showing the mean (± S.E.M; *n* = 8 cells) and individual amplitude of I-instant (I-inst.; **B**) and I-steady **(C)** during treatments with KT5720, KT5720 + GLP-1, and washout of GLP-1; Right panel showing the mean (± S.E.M; *n* = 6 cells) and individual amplitude of I-instant (I-inst.; **B**) and I-steady **(C)** during treatments with H89, H89 + GLP-1, and washout of GLP-1 (Right panel). **(D,E)** Left panel showing the mean (± S.E.M; *n* = 8 cells) and individual amplitude of IH **(D)** and IT **(E)** during treatments with KT5720, KT5720 + GLP-1, and washout of GLP-1; Right panel showing the mean (± S.E.M; *n* = 6 cells) and individual amplitude of IH **(D)** and IT **(E)** during treatments with (left panel); H89, H89 + GLP-1, and washout of GLP-1 (Right panel).

Activation of G protein-coupled receptors can regulate the functional activities of IH channels through cAMP, cGMP, and/or Ca^2+^ signaling pathways (Pape, 1996; Ludwig et al., 1998; Biel et al., 2002). We therefore used a selective PKA inhibitor, KT5720 (500 nM), and a general PKA inhibitor, H89 (10 μM), to examine whether the effect of GLP1 on IH channel activity occurs through activation of PKA. To inhibit PKA, cerebellar slices were incubated for 20 min in ACSF containing KT5720 or H89 before patch-clamp recording. In the absence of PKA activity, application of GLP1 failed to increase hyperpolarization-elicited membrane currents (Figure 9A). In the presence of KT5720 and GLP1, the mean value of I-instant was 1156.9 ± 10.8 pA, which was similar to that of the control (KT5720; *p* > 0.05, *n* = 8, Figure 9B). The mean value of I-steady was 238.9 ± 11.1 pA, which was not significantly different from that of control (KT5720; *p* > 0.05, *n* = 8, Figure 9C). The mean value of IH was 82.0 ± 7.3 pA, which was not significantly different from that of the control (KT5720; *p* > 0.05, *n* = 8, Figure 9D). The mean value of I-tail was 33.9 ± 2.0 pA, which was similar to that of the control (KT5720; *p* > 0.05, *n* = 8, Figure 9E). In the presence of H89 and GLP1, the mean value of I-instant was 1164.3 ± 10.0 pA, which was similar to that of the control (H89; *p* > 0.05, *n* = 8, Figure 9B). The mean value of I-steady was 243.2 ± 7.1 pA, which was not significantly different than that of the control (H89; *p* > 0.05, *n* = 8, Figure 9C). The mean value of IH was 81.5 ± 6.5 pA, which was not significantly different from that of the control (H89; *p* > 0.05, *n* = 8, Figure 9D). The mean value of IT was 33.7 ± 2.8 pA, which was similar to that of the control (H89; *p* > 0.05, *n* = 8, Figure 9E). These results indicate that GLP1 enhanced the activity of IH channels through activating PKA, and that GLP1 facilitates IH and increased excitability of PCs through its receptor and PKA signaling.

## 4. Discussion

Our results show that GLP1 depolarized the membrane potential and increased the spike firing rate, but significantly inhibited the after-hyperpolarizing potential and outward rectifying current of PCs *via* GLP1 receptors. Furthermore, GLP1 significantly enhanced the hyperpolarized stimulation-evoked instant current, steady current, I-tail and IH currents, which was abolished by the selective IH antagonist, ZD7288. Moreover, the GLP1-induced enhancement of membrane currents was abolished by a selective GLP1 receptor antagonist, as well as by inhibition of PKA. These results indicate that GLP1 receptor activation enhances IH channel activity *via* PKA signaling, resulting in increased excitability of mouse cerebellar PCs *in vitro*.

### 4.1. GLP-1 enhances the excitability of cerebellar PCs through its receptors

In the cerebellum, GLP1 receptors are abundant in cerebellar PCs (Diz-Chaves et al., 2020). Our present results are consistent with those of a previous study (Diz-Chaves et al., 2020); GLP1 receptor immunoreactivity was present in the cerebellar cortex and was abundant in PCs, indicating that GLP1 signaling might play an important role in regulating PC activity (Farkas et al., 2021). We showed that after blockade of glutamate and GABAergic synaptic transmission, GLP1 and its analogs increased the excitability of PCs, indicating that GLP1 enhances the excitability of PCs through a postsynaptic signaling pathway.

Early studies demonstrated that activation of GLP1 receptor in rat hippocampal CA1 neurons regulated the spike firing activity of neurons through synaptic transmission mediated by non-NMDA glutamate receptors (Oka et al., 1999). GLP1 inhibits or excites neurons expressing its receptor in the mouse bed nucleus of the striaterminalis, 40% of which show excitation, and 60% of which show inhibition (Williams et al., 2018). In addition, GLP1 induces depolarization in most hippocampal neurons and hyperpolarization in a few neurons (Cork et al., 2015). In the hypothalamic paraventricular nucleus, activation of GLP1 receptor on CRH neurons up-regulates the function of AMPA receptors through PKA signaling, and enhances excitatory glutamate synaptic transmission (Liu et al., 2017). In the arcuate nucleus, GLP1 analogs act directly on the postsynaptic receptors, leading to membrane depolarization, accompanied by enhanced spontaneous action potential activity (Secher et al., 2014; He et al., 2019). Furthermore, GLP1 induces depolarization of ganglion cell resting membrane potential through the cAMP pathway, accompanied by an increase in intracellular Ca^2+^ concentration, leading to a significant increase in spike firing frequency (Kakei et al., 2002). Moreover, GLP1 receptor activation increases the spike firing frequency of gonadotropin-releasing hormone neurons in male mice and significantly increases the frequency of miniature synaptic postsynaptic currents, indicating that GLP1 receptor activation promotes GnRH neuronal activity (Farkas et al., 2016). Consistent with previous studies (Kakei et al., 2002; Farkas et al., 2016; Liu et al., 2017), we found that GLP1 and its analogue significantly increased the spike firing frequency of PCs in the absence of inhibitory and excitatory synaptic afferents, indicating that GLP1 receptor activation enhances neuronal excitability, leading to membrane depolarization and an increased spike firing rate in cerebellar PCs.

#### 4.1.1. Mechanism of GLP1-enhanced spike discharge activity in PCs

The simple spike discharge activity of cerebellar PCs is related to the activities of outward rectified potassium channels, non-selective cation channels, and IH channels (Eccles et al., 1966; Ito, 1984, 2006; Bear et al., 2007; Light et al., 2015; Hoxha et al., 2018). We showed that GLP1 significantly inhibited outward rectified potassium ionic currents across the PC membrane, indicating that GLP1 suppressed the amplitude of the after-hyperpolarization potential and accelerated repolarization *via* its receptor. The IH ion channel is key for autonomic activity of neurons, and plays a key role in controlling the periodicity of continuous and intermittent action potential firing and stability of the membrane potential (Maccaferri and McBain, 1996; Ghamari-Langroudi and Bourque, 2000; Southan et al., 2000). In the cerebellar cortex, PCs are autonomous cells, and the IH channel plays a key role in the simple spike discharge activity (Crepel and Penit-Soria, 1986; Roth and Häusser, 2001; Shim et al., 2016). We showed that GLP1 reversibly increased the IH current, indicating that GLP1 up-regulated IH channel activity. Blocking the IH channel or GLP1 receptor completely eliminated the GLP1 enhancement of the PC membrane current, which indicated that GLP1 enhanced the activity of IH channels and increased the excitability of cerebellar PCs by activating GLP1 receptor.

GLP1 receptor is a G-protein-coupled receptor that is widely expressed in the paraventricular nucleus, arcuate nucleus, ventral tegmental area, nucleus tractus solitaries, and cerebellum (Campos et al., 1994; Dunphy et al., 1998; Pyke et al., 2014; Cork et al., 2015; Heppner and Perez-Tilve, 2015; Jensen et al., 2018). GLP1 receptor is considered to be excitatory and to play critical roles in regulating neuronal activity and synaptic transmission *via* cellular signaling pathways (Hällbrink et al., 2001; Cork et al., 2015; Williams et al., 2018). A variety of neurotransmitters combine with G protein-coupled receptors to modulate the activities of IH channels by regulating the production of cAMP (Pape, 1996; Frère and Lüthi, 2004). Activation of GLP1 receptor can increase the level of cAMP by activating adenylate cyclase, thereby modulating signal transduction and the activity of IH channels (Pape, 1996; Ludwig et al., 1998; Biel et al., 2002; Mayo et al., 2003; Shim et al., 2016). Our results show that GLP1 failed to increase hyperpolarization-elicited membrane currents in the absence of PKA activity, which indicated that GLP1 enhanced the activity of IH channels through PKA signaling. In addition, GLP-1 induced changes of membrane potential and firing rate may be affected by injecting negative currents or positive square pulses. However, these injected currents were maintained constant throughout the experiments, suggesting they should not significantly change the GLP-1-induced depolarization. Taken together, the present results are consistent with previous studies (Dautzenberg et al., 2000; Dautzenberg and Hauger, 2002; Grammatopoulos and Chrousos, 2002; Facci et al., 2003), indicating that GLP1 binds with its receptor and enhances the activation of adenylate cyclase, thereby increasing the level of intracellular cAMP and the activity of PKA, resulting in increased excitability of mouse cerebellar PCs *in vitro*.

### 4.2. Physiological significance of GLP1; modulation of spike discharge activity of PCs

The cerebellum plays a key role in motor regulation and coordination (Ito, 1984, 2006). PCs are the sole output cells of the cerebellar cortex and their axons project to the deep cerebellar nucleus. PCs are GABAergic neurons that exhibit highly autonomic simple spike discharge activity and inhibit the activity of deep cerebellar nucleus neurons by the release of GABA (Ito, 1984, 2002). We show abundant GLP1 receptor immunoreactivity in the PCs of the cerebellar cortex (Diz-Chaves et al., 2020; Farkas et al., 2021) and that GLP1 combines with its receptor to activate IH channels through PKA signaling. This result in PC membrane depolarization and an increase in spike firing rate. In addition, the cerebellar cortex receives preglucagonergic neuron projections (Campos et al., 1994; Cork et al., 2015), which indicates that endogenous GLP1 may be released and participate in motor regulation and coordination by increasing spike firing activity of PCs. Together, the present results provide novel insight into the function of GLP1 in the central nervous system and indicate that GLP1 modulates motor coordination and motor learning by increasing cerebellar PC output.

## Data availability statement

The original contributions presented in the study are included in the article/Supplementary material, further inquiries can be directed to the corresponding authors.

## Ethics statement

The animal study was reviewed and approved by the Animal Care and Use Committee of Yanbian University.

## Author contributions

D-LQ, C-PC, and Y-HB conceived and designed the experiments. YL, L-XC, W-YW, and Y-RP performed the electrophysiological experiments. J-YW performed immunohistochemistry experiments. C-PC and YL analyzed the data. C-PC, YL, and D-LQ wrote the manuscript. All authors contributed to the article and approved the submitted version.

## Funding

This work was supported by the National Natural Science Foundations of China (32070986, 32171005, and 32260195), the Major Projects of the Ministry of Science and Technology of China (2021ZD0202300), and the Key Projects of Science and Technology Development Plan of Jilin Province, China (20210204152YY).

## Conflict of interest

The authors declare that the research was conducted in the absence of any commercial or financial relationships that could be construed as a potential conflict of interest.

## Publisher’s note

All claims expressed in this article are solely those of the authors and do not necessarily represent those of their affiliated organizations, or those of the publisher, the editors and the reviewers. Any product that may be evaluated in this article, or claim that may be made by its manufacturer, is not guaranteed or endorsed by the publisher.

## Supplementary material

The Supplementary material for this article can be found online at: https://www.frontiersin.org/articles/10.3389/fnmol.2023.1126447/full#supplementary-material
